# System analysis of the fast global coronavirus disease 2019 (COVID-19) spread. Can we avoid future pandemics under global climate change?

**DOI:** 10.1080/19420889.2022.2082735

**Published:** 2022-05-30

**Authors:** Vadim Volkov

**Affiliations:** Research Institute of Russian Academy of Sciences, K.A. Timiriazev Institute of Plant Physiology RAS, Moscow, Russia

**Keywords:** global climate change, COVID-19, system approach, zoonosis, pandemic

## Abstract

The recent fast global spread of COVID-19 caused by a severe acute respiratory syndrome coronavirus 2 (SARS-CoV-2) questions why and how the disease managed to be so effective against existing health protection measures. These measures, developed by many countries over centuries and strengthened over the last decades, proved to be ineffective against COVID-19. The sharp increase in human longevity and current transport systems in economically developing countries with the background of persisting cultural frameworks and stable local pools of high bacterial and viral mutations generated the wide gap between the established health protection systems and the new emerging diseases. SARS-CoV-2 targets human populations over the world with long incubation periods, often without symptoms, and serious outcomes. Hence, novel strategies are necessary to meet the demands of developing economic and social environments. Moreover, the ongoing climate change adds extra challenges while altering the existing system of interactions in biological populations and in human society. Climate change may lead to new sources of viral and microbial mutations, new ways of zoonotic disease transmission and to huge social and economic transformations in many countries. The present short Opinion applies a system approach linking biomedical, climate change, social and economic aspects and, accordingly, discusses the measures and more efficient means to avoid future pandemics.

## Introduction

The accelerated frightening and unexpected global distribution of COVID-19 with over 170 million infected worldwide by June 2021, 240 million by November 2021 and over 470 million by April 2022 with mortality over 2.1%, 2.0% and 1.3% at the times, correspondingly (data from https://gisanddata.maps.arcgis.com/apps/opsdashboard/index.html#/bda7594740fd40299423467b48e9ecf6 equivalent to https://coronavirus.jhu.edu/map.html and https://covid19.who.int/) is caused by RNA virus SARS-CoV-2 from *Coronaviridae* family [1,2]. The genome of the virus is about 30,000 nucleotides with a high rate of mutations at hotspots of the genome which helped to trace the viral distribution and origin from country to country around the world (e.g [3], based on GISAID database https://www.gisaid.org/phylodynamics/global/nextstrain/ [4]). The pandemic situation was classified as a “a once-in-a-century” event (e.g [5]) when the death toll was comparable to or sharply exceeded all the major battles or causalities after the World War 2 (e.g [6]).

The main questions emerging from the start of the COVID-19 pandemic are about the origin of the disease, its unusual features and the reasons for the unexpectedly fast spread of the COVID-19. Moreover, we have to consider and develop new strict, robust, and reliable measures to prevent future potential expected pandemics under the situation of the global climate change.

## Start and the sources of the COVID-19 pandemic

The COVID-19 pandemic started in Wuhan province in China [1,7,8] and was initially linked to a local market where the illegal sales of wild animals and seafood took place [1,7]. Interspecies zoonotic viral transmission from bats to humans seems the most likely trigger of the pandemic [1]. Several sources attributed the origin of SARS-CoV-2 to populations of *Rhinolophus macrotis* bats which serve as reservoir host for the virus (cited in [9]). The similarity between human SARS-CoV-2 and coronaviruses in the bat *Rhinolophus affinis* was initially determined as high as 93.3% (cited in [9]) (though this is lower than the similarity between the genomes of human and chimpanzee; 96–99% [10]). However, coronaviruses similar to SARS-CoV-2 have been found in other bat species and in pangolins from different locations in South-East Asia [11–13]. Further analysis of SARS-CoV-2 from humans and other isolated viral sequences from *Rhinolophus affinis* indicated a similarity of 96.2% [14]. Taken together, the sequence and contact data strongly support the natural (possibly multiple) zoonotic origin of SARS-CoV-2 [15,16] though laboratory intervention cannot be completely excluded. The high probability of new unlimited pools of emerging viral infections is confirmed by, for example, newly characterized Nipah viruses from bats which belong to zoonotic RNA-negative viruses of the *Paramyxoviridae* family and are associated with recent near-annual outbreaks in Bangladesh and India with over 70% mortality in humans [17,18].

It is worth mentioning the recent earlier zoonotic SARS-CoV spillover in 2002–2003 which was localized mostly in the South-East Asia. The first cases of an unknown disease caused by coronavirus SARS-CoV started in Guandong Province of southern China (nearly 1000 km south of Wuhan) and spread to many countries (more information and references are in [19]). The infection was restricted finally to 29 countries who informed the World Health Organization of over 8,000 SARS cases with 774 deaths [20]. Later, several sporadic cases of SARS-CoV were registered again in Guangdong Province, China in 2003 [19]. In 2003, SARS-CoV-like viruses (up to 99.8% homology) were isolated from palm civets from the live-animal market in Guandong with evidence for the infection in raccoon dogs and in humans working at this market [21].

## Biomedical aspects of COVID-19 which promoted pandemic and hampered the detection of the infection

The ancestral viral SARS-CoV-2 strains cause the complex of several characteristic features of COVID-19 which led to the pandemic. The features include:

1) incubation period of several days to over two weeks for the infection (e.g [22,23]);

2) age-dependent susceptibility to the infection (e.g [24] halving for ages below 20 years old with specific age-dependent correlations);

3) age-dependent severity and mortality for the infection (both essentially increase for ages over 60 years) ([22] data and citations from [25];

4) gender-dependent severity (more severe symptoms for male) of the infection (e.g [22,26]);

5) asymptomatic infection for 40–45% of infected persons but who can spread the virus over 14 days and longer [27].

The median incubation period (e.g [22,23]) of COVID-19 is 4.8 days, with 95% of those infected having developed symptoms by the 14th day of infection [22]. Over most of this prodromal period, the infected persons can transmit SARS-CoV-2 to persons with whom they come in contact. Therefore, the period was quite important to localize the infection, it requires highly sensitive molecular methods of reverse-transcription polymerase chain reaction (RT-PCR) to detect RNA of SARS-CoV-2. Here is one of the reasons why COVID-19 was not known decades ago before advance in molecular methods. The incubation periods for most acute respiratory viral infections are much shorter being 1–2 days for commonly known influenza and rhinovirus [28]. A longer incubation period, similar to COVID-19, is characteristic of diseases such as measles [28] where immunization of children aged 12–23 months reached over 85% in the world in 2019 (https://data.worldbank.org/indicator/SH.IMM.MEAS). It could be one more reason for the spread of COVID-19 – a general reduced attention to diseases with long incubation periods.

**Figure 1.**
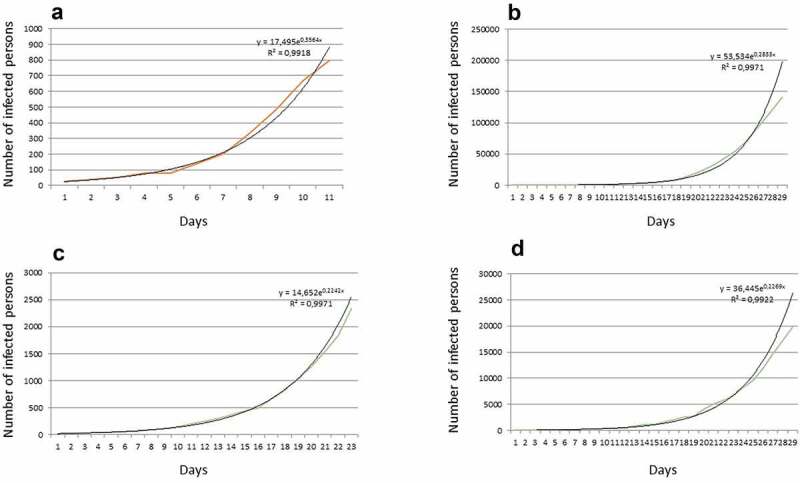
Number of revealed infected persons at the initial stage of COVID-19 spread for several countries with different size and political background: (a) Luxembourg, (b) USA, (c) Russia, (d) UK. The color lines are the original data, the black lines are the exponential fits. The initial dates are chosen arbitrary based on the start of COVID-19 spread in the mentioned countries in the spring of 2020.

The severity of COVID-19 caused by ancestral viral strains increases with the age of the infected person, especially for those over 60 years of age ([22] data and citations from [25]) who often suffer from other chronic diseases. Hence, the clinical symptoms of COVID-19 again could be masked. Lower susceptibility to COVID-19 (for initial SARS-CoV-2 strains) of younger (especially below 20 yo) persons (e.g [24]) and asymptomatic infections [27] add more complexity for detection and control of the disease. The age-related and gender-related differences could be explained by corresponding distinct different patterns of expression of angiotensin-converting enzyme 2 (ACE-2) which also serves as a receptor for several coronaviruses [29], so seems to be naturally predictable. The more convoluted explanations are expected since cell binding and proliferation of SARS-CoV-2 involves multiple steps with over 330 specific protein-protein interactions [30] and over 100 RNA-protein interactions [31]. It is also essential to mention that some of the later SARS-CoV-2 strains (e.g. Delta variant) that evolved within the pandemic have much higher transmissivity, indications of higher severity and differ in COVID-19 caused symptoms (e.g [32–34]). The distribution of Delta variant of SARS-CoV-2 coincided with sharp increase of COVID-19 cases in children and adolescents being often without previous age-dependency (e.g [35]). Fast exponential increase in the number of infected persons at the initial stage of COVID-19 spread was typical for most countries (https://gisanddata.maps.arcgis.com/apps/dashboards/bda7594740fd40299423467b48e9ecf6; Figure 1), definitely indicating that the existing common disease protection measures were not sufficient to control this infection. The exponential initial curve was similar for countries with different sizes of population and political backgrounds.

## New social and economic factors which promoted the COVID-19 pandemic

It is important to determine the factors that influenced the global spread of COVID-19 from the very start of the infection in Wuhan [1,7,8]. The average length of life has sharply increased in China, more than doubling within the last 70 years: it was 35 years before 1949, increasing to 76.3 years in 2015 (cited according to [36]). The increase is associated mainly with the extended duration of individual lives [36] though a sharp decrease of infant mortality from 320/1000 births in 1950 to 12/1000 births in 2015 also contributed (https://www.statista.com/statistics/1041851/china-all-time-child-mortality-rate/). The change developed within 2–3 generations and overlapped the booming rise, over 10 times, of fast transport infrastructure in China [37] and global international air transport. Consequently, the local population from Wuhan, traveling worldwide, became the global carriers of the previously unknown COVID-19 which had not previously widely emerged in China due to the shorter length of life (disease is expressed much more strongly for older persons, see above), lower level of medicine and relatively low mortality of the disease, typically below 3–5%.

At the same time, cultural frameworks [38] kept for generations over centuries in many countries, including China, form the milieu and social and cultural background to the spread of pandemics. The seafood markets with wild animals for food consumption, large food courts and close communications were the cultural peculiarities which overlapped with social changes, economic growth and biological mutating pools of infection. The combination of factors promoted the COVID-19 pandemic.

From the global point of view, the rise of world population, population density growth with an increase in percentage of urban population, especially in the developing countries, facilitated the transmission of COVID-19. It is also likely that the main part of the human population over the world never previously encountered similar coronavirus infections and so SARS-COV-2 had more severe consequences than in Wuhan. More specific innate immunity [25] could be formed over centuries in the local areas of China and East Asia where the natural reservoir of host animals for these coronaviruses live. Indeed, the host genetic factors are important for COVID‐19 susceptibility and severity [39]. Indirectly supporting this view is the observation that over 80% of individuals in Wuhan (532 in a study of 9542) with pan-immunoglobulins against SARS-CoV-2 had asymptomatic infection while the number is only 40–45% worldwide [40]. The question, however, requires more investigation.

It is also reasonable to compare the present pandemic caused by SARS-CoV-2 to the mentioned recent disease caused by SARS-CoV. At the time (2002–2003) transport systems were less developed, there was lower GDP per capita in China (which correlated with lower travel activity) and less global air transport (over 2.5 lower) (https://data.worldbank.org/indicator/IS.AIR.PSGR). These factors together with the much more severe effects of the SARS-CoV infection (which led to initial sharper measures) than for SARS-COV-2 are the likely reasons why a pandemic situation was avoided in 2002–2003.

## Brief summary on climate change

Ongoing climate change within the 20th century is following a documented rise of CO_2_ concentration in the Earth’s atmosphere from below 300 ppm to 390 ppm and doubling of methane concentration to nearly 1800 ppb, all leading to the global mean surface temperature increase by about 0.6°C with higher speed over the last decades (e.g [41–43]). The main source of the changes is human activity leading to CO_2_ and methane emissions by industry and intensive agriculture [44], which has expanded to feed the rapidly growing human population [45]. Several scenarios are considered for the 21st century: the climate warming is expected to be between 1°C and 3.7°C depending on greenhouse gas emissions [42]. Each of the scenarios, however, has more or less devastating effects on terrestrial and ocean ecosystems and particularly on countries with maritime coastal areas.

The temperature increase causes melting of polar ice caps and rise of water level in oceans, by about 20 cm in the 20th century (depending on method of estimates [44]). The average observed rise of temperature by 0.6°C is more reflected in higher frequency and stronger expression of extreme events (heat waves, warm spells, floods, forest fires, droughts, precipitation extremes etc.) (e.g [46]). In Europe, the recent consecutive summer drought of 2018–2019 is unprecedented in the last 250 years [47] while the heatwave of 2003 was the hottest over the last 500 years and increased mortality by around 40,000, who were mostly elderly persons [48]. The explanation lies in highly nonlinear responses of many complex physical systems including Earth’s climate system (e.g [49]).

## Amplification of biological and social factors promoting potential pandemics by the ongoing climate changes

From the point of biology, the climate change leads to increased rate of mutations and evolutionary changes in adapting populations (e.g. reviewed in [50–52]) followed by nearly unlimited rate of corresponding viral and bacterial mutations. The climate change also modifies the behavior of wild animals as clearly shown for Arctic species [53], further it may potentially alter the trophic chains and transmission of diseases. The effects of climate change on trophic efficiency of food chains are being investigated (e.g [54,55]). Evidently, the efficiency of energy and trophic biomass transfer is among the major factors determining the stability and length of trophic chains (e.g. reviewed in [55,56]). Hence, the climate change can restructure the trophic chains while modifying the complex pattern of viral and bacterial transmissions. Finally, it may result in new ways of disease transmissions with higher rates of mutations. This is a serious threat to our current epidemiological knowledge. Although still more speculative, the suggestions find more support and experimental evidence.

Predictably the areas where new pandemics may originate are the developing countries of South East Asia, Latin America and Africa with growing population density, developing transport infrastructure, enlarging flows of tourists, and unexplored natural pools of viral and microbial infections. The existence of obsolete cultural frameworks (a sharp example, is the consequences of cannibalism in some culturally closed areas of Africa (e.g [57]) and at certain isolated islands in Pacific Ocean, which are slowly being eradicated) and fast economic development can be superimposed on the climate changes.

The rise of sea level has a great impact and leads to flooding of vast territories, salinization of agricultural lands, shortage of freshwater and desertification (e.g [58]). It is estimated that about 200 to 630 million people currently live in areas below high tides lines expected by 2100 under low and high carbon emissions, correspondingly [59]. The combination of mentioned factors including heatwaves facilitates the emergence and spread of new infections, especially in developing countries where increase in population density, malnutrition, limited access to freshwater, further use of wild animals for food are difficult to control and alleviate under the circumstances.

It should be mentioned, however, that we still are not aware of many factors which may influence the pandemics of different origin and revealing unexpected correlations from the present COVID-19 pandemic. Recent findings demonstrated that it is the latitude of countries linked to the sun UV daily dose but not temperature and humidity that strongly correlated with dates of autumn surges of COVID-19 [60]. The expected contributing factor was vitamin D [60]. It is also suggested that potassium balance surprisingly influences COVID-19 severity [61]. These factors are likely not influenced by the ongoing climate changes though add complexity to the analysis and modeling.

## Discussed measures to prevent future potential expected pandemics

**Figure 2.**
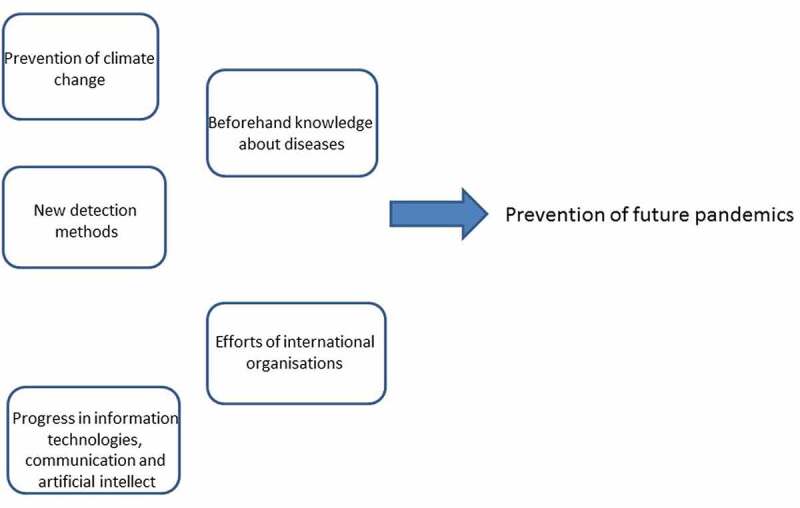
The scheme for the system of measures against the future pandemics.

The present situation with exponential economic and population growth in developing and not-well-explored areas of our planet combined with the development of global transport infrastructure is opening a Pandora’s box of new and unexpected potential pandemics. Global climate warming fixes the point of no return with several scenarios and lack of determined predictability. A simple rather evident scheme of a few clear measures is represented here (Figure 2) with an optimistic hope that there are still opportunities to prevent pandemics and to control them (if any) employing new biomedical technologies, a holistic approach to our civilization and amended uses of information technologies to improve communication, health management, and regulations.

Climate change is a major concern for low predictability since we consistently see new harmful and potentially disastrous consequences of climate change (see above: desertification, land salinization, threat to agriculture with consequent malnutrition for many countries, expected flooding of populated areas with further unavoidable migration of the populations up to hundreds of millions; exposure to new dormant and unknown infections from natural sources due to changes in animal behavior and in trophic chains; expected rise of viral and microbial mutations due to their new hosts and conditions). The common goals to reduce greenhouse gas emissions, use green energy, and stop the climate warming are evidently not yet sufficient.

The bright side comes from the new faster, cheaper and reliable molecular technologies and tests (modifications of PCR, antibody-based methods, DNA and RNA sequencing). For example, fast isothermal PCR with portable systems (e.g [62]) could be a candidate for express-tests within 30 minutes to 1 hour, while more advanced laboratory-based methods of cellular, protein and DNA and RNA sequencing could be developed for multiple clinical purposes. The present anti-pandemic measures on airlines could be applied wider with protection masks, free PCR-tests shortly in advance and after the travels and the fast results, which are transferred to the passengers, transport providers, and global databases.

The availability of the www across the world, immediate information exchange, new artificial intelligence (AI) systems that aid to detect infected persons, alarm about new sources of infections provide help in predicting the spread of infections. The obvious requirement in preventing future diseases and controlling them is prior knowledge. Essential efforts in development of new broader vaccines and technologies for their fast production together with global access to the vaccines are the further steps to prevent the spread of potential pandemics [63]. The present situation clearly demonstrated that poor funding of research may lead to disastrous consequences for world economy, the initial estimates that the costs of COVID-19 pandemic would exceed $ 16 trillion [64] could be a lower level of real losses.

New knowledge and tools will not be sufficient against pandemics without the common efforts of all countries and the international organizations (UN https://www.un.org/en/, WHO https://www.who.int/, FAO http://www.fao.org/about/en/, UNESCO https://en.unesco.org/ etc.) whose growing role and influence are required to negotiate and find appropriate solutions.

Evidently, the outlined measures are not comprehensive and not depicted in detail. However, the sketch could be a backbone or part of it for future development.
